# Effects of Structural Factors of Hydrated TiO_2_ on Rutile TiO_2_ Pigment Preparation via Short Sulfate Process

**DOI:** 10.1038/s41598-020-64976-4

**Published:** 2020-05-14

**Authors:** Congxue Tian

**Affiliations:** 1 Panzhihua University, Panzhihua, 617000 Sichuan, China; 2 Key Laboratory of Green Chemistry of Sichuan Institutes of Higher Education, Zigong, 643000 Sichuan, China

**Keywords:** Chemistry, Materials science

## Abstract

The structural factors such as crystal structure, particle size distribution and impurity content of hydrated TiO_2_ had great effects on the structures and pigment properties of the rutile TiO_2_. The rutile TiO_2_ white pigment was prepared via the Short Sulfate Process from low concentration industrial TiOSO_4_ solution. In order to produce rutile TiO_2_ pigment with good structures and excellent pigment properties, the crystal size of the hydrated TiO_2_ should be controlled less than 8.9 *nm* and as close as possible to 7.9 *nm*, which could effectively promote the phase transformation and crystal growth of the rutile TiO_2_. The appropriate particle size distribution of hydrated TiO_2_ had obvious effects on obtaining rutile TiO_2_ with narrower particle size distribution and near 0.20 *µm*. It was best to adjust the hydrolysis conditions to reduce the specific surface area of the hydrated TiO_2_ so as to reduce the iron ion impurity adsorption.

## Introduction

In the material science, the material’s properties are determined by their structural factors, such as the crystal structure, phase composition, chemical composition, and so on, its essence is the reflection of material structure theory. And the structural factors would be determined by the preparation process and controlling conditions, which would ultimately determine the properties and applications of the materials. Titanium dioxide (TiO_2_) is the third largest commercial inorganic chemicals, either in anatase or rutile polymorphs, and due to its excellent properties such as higher refractive index, smaller crystal size, better optical properties and chemical inertness, it has become the most widely used white pigments, used in many fields such as coatings, paints, paper, fibers, cosmetics, sunscreen products, chemical catalysis, electronic materials and so forth^1–3^. Titanium dioxide white pigment is commercially prepared by either chloride process or sulfate process. In recent year, the market demand of titanium dioxide increases by about 3% every year. In 2019, the global titanium dioxide production capacity was 8,685,000 tons, the production capacity was of 3,845,000 tons and the output was of 3,137,200 tons in China in 2019, and the output of the sulfate process accounting for 93.6% in China. The core step for titanium dioxide production via the sulfate process is the hydrolysis of the titanyl sulfate solution. The hydrolysis process would undergo a series of complex physical and chemical reactions, and the hydrolysis parameters and conditions would have great effects on the structure of the hydrolysis products (hydrated TiO_2_, also named metatitanic acid), and the structure and quality of hydrated TiO_2_ would ultimately determine the structure and properties of the titanium dioxide pigment. And the thermal hydrolysis reactions of industrial TiOSO_4_ solution include nucleation, crystal growing, polymerization, agglomeration, aggregation and precipitation, accompanied by hydrolysis precipitation of crystalline TiO^2+^ ions via olation and oxolation reaction^4^. Researches show that the hydrated TiO_2_ is colloidal particles, formed by nuclei growing and aggregating, and eventually forming the secondary aggregated particles with the particle size of 10–200 *μm*^5,6^. Many researchers have widely investigated the hydrolysis process and conditions, effects of the additives and hydrolysis parameters on the products, as well as properties and applications of titanium dioxide^7–16^. Urakaev *et al*. investigated the homogeneous nucleation and growth of monodispersed spherulites of sulphur and hydrated titanium dioxide of anatase modification by a new coherent optical method for measurement of the relative scattering coefficient based on alternative use of laser radiation of various wavelengths^17^. Ultrasonic technology was used to pretreat hydrated TiO_2_ slurry before washing, which showed that the removal effect of iron impurities in hydrated TiO_2_ was better and the effect of washing water saving was obvious, while the lattice parameters of TiO_2_ had no effect on rutile and product quality^18^. The effects of ball milling process parameters and stress energy on the particle size distribution of TiO_2_ were investigated, and the results showed that particle size distribution had greatly influenced the pigment performances^19^. The particle size and its distribution of TiO_2_ had prominently affected its spectral reflectance and color coordinates, and a model was established to investigate the effect of particle size on the aesthetic and thermal properties of poly-dispersed titanium dioxide pigments coatings^20^. By investigating the precipitation and growth behavior of hydrated TiO_2_ hydrolyzed from titanyl sulfate solution, the hydrolysis temperature was the most important factor affecting the particle size of hydrated TiO_2_, and the hydrated TiO_2_ particles were easy to aggregate in the preparation process, which could be described by an empirical expression^21^. The physicochemical properties of anatase TiO_2_ nanoparticles could also be changed by using surface treatment^22^. However, there were few reports about the effects of the structural factors of hydrated TiO_2_ on the pigment properties. The short sulfate process refers to the titanium dioxide preparation by using unenriched low concentration TiOSO_4_ solution as titanium source via sulfate process, cancelling the concentration section of diluted TiOSO_4_ solution, having the advantages of short process, low cost and low energy consumption, which could promote the technological innovation of traditional sulfate process for TiO_2_ pigment production^23,24^.

The structures of the hydrated titanium dioxide had great impacts on TiO_2_ production. Herein, hydrated TiO_2_ was prepared via short Sulfate Process by using the unenriched low concentration TiOSO_4_ solution as raw material to produce rutile TiO_2_ pigment. It was important to investigate the influences of the crystal structure, particle size distribution and impurity of the hydrated TiO_2_ on the TiO_2_ pigment preparation.

## Experimental

Rutile TiO_2_ pigments were prepared from different low concentration industrial TiOSO_4_ solution (the total TiO_2_ concentration ranging from 155 *g/L* to 180 *g/L*, weight concentration) as titanium sources, through thermal hydrolysis by authigenic seed method via the Short Sulfate Process. The typical hydrolysis process was carried out as listed in our literature^23^, and the pre-adding water volume ratio (as water to TiOSO_4_ solution) was of 0.18:1, the hydrolysis time after the second boiling point was of 2.5 *h*, then finished the hydrolysis process and obtained the hydrated TiO_2_. The as-prepared hydrated TiO_2_ was washed with water, bleached and filtered, then whipped to slurry with the deionized water uniformly. The slurry was doped with the rutile calcining seed (5%, as to TiO_2_, *wt* %), zinc salt (ZnO of 0.26%), potassium salt (K_2_O of 0.50%) and phosphate salt (P_2_O_5_ of 0.11*%*), then calcined in a muffle furnace in the air atmosphere. The calcining conditions was as the following: firstly from room temperature raising to 420 °*C* in 60 *min* and holding for 30 *min* at 420 °*C*, secondly from 420 °*C* to 780 °*C* in 60 *min* and holding for 60 *min* at 780 °*C*, and lastly from 780 °*C* to 870 °*C* in 120 *min* and holding for 40 *min*. Then the rutile TiO_2_ pigment powder was obtained after cooling and grinding by the three head grinder. The different concentration of industrial TiOSO_4_ solution was conducted at 155 *g/L*, 161 *g/L*, 167 *g/L*, 173 *g/L*, 180 *g/L*, and the obtained hydrated TiO_2_ samples were marked as A, B, C, D, E, the rutile TiO_2_ samples after salt treatment and calcination were denoted as A1, B1, C1, D1, E1, respectively.

The crystal structures of hydrated titanium dioxide and rutile TiO_2_ were determined by the XRD analysis (X’ Pert3 Powder, PANalytical), and the crystal size *L*_*(101)*_ for the anatase crystal plane *(101)* of hydrated TiO_2_ and *L*_*(101)*_ for rutile TiO_2_ crystal plane *(110)* was calculated according to Scherrer equation (Eq. A), where *K* was the constant (0.8900), *λ* was the wavelength of Cu*Kα*_*1*_ (0.15418 *nm*), *β* was the full width at half maximum intensity (FWHM) of crystal plane for XRD peak in radians, and *θ* was the Bragg’s diffraction angle, respectively. The rutile content (*X*_R_) was calculated according to Eq. B, where *I*_*A*_ and *I*_*R*_ represented the integrated intensity of the anatase (101) main peak and the rutile (110) main peak, respectively.

A $${\rm{Crystal}}\,{\rm{size}}:\,L=K\lambda /\beta \cdot \,\cos \,\theta $$

B $${\rm{Rutile}}\,{\rm{phase}}\,{\rm{content}}:\,{X}_{R}={I}_{R}/0.884{I}_{A}+{I}_{R}$$

Particle size distribution (*PSD*) test was carried out on a Malvern particle size analyzer (Malvern Zetasizer Nano ZS90). The specific surface area of hydrated TiO_2_ was measured on the surface and pore size distribution instrument (3H-2000PS1, Beishide, China). The *S*_*BET*_ of the hydrated TiO_2_ samples were calculated by the BET multi-point method according to the N_2_ adsorption-desorption curves. The UV–vis diffuse reflection spectra were obtained on a ultraviolet visible spectrophotometer with integral ball accessories (U-4100, Hitachi). The particle morphology was observed on a JEOL scanning electron microscopy (JSM-7100F). The surface morphology was carried out on a field emission transmission electron microscopy (Tecnai G2 F20S-TWIN) at 200 kV. The impurities of the rutile samples was determined on an ICP-AES (ICAP 6300, Thermo Scientific Co. Ltd). The ultra-precise colorimeter (LabScan EX, American Hunter) was used to determine the pigment properties, such as the chromatic power (*TCS*), blue phase (*SCX*), the brightness (*Jasn*) and the relative scattering force (*Rs*), by using the R930 (Ishihara Sangyo Kaisha, Ltd.) as the standard reference sample.

## Results and Discussions

### Crystal structure

**Figure 1:**
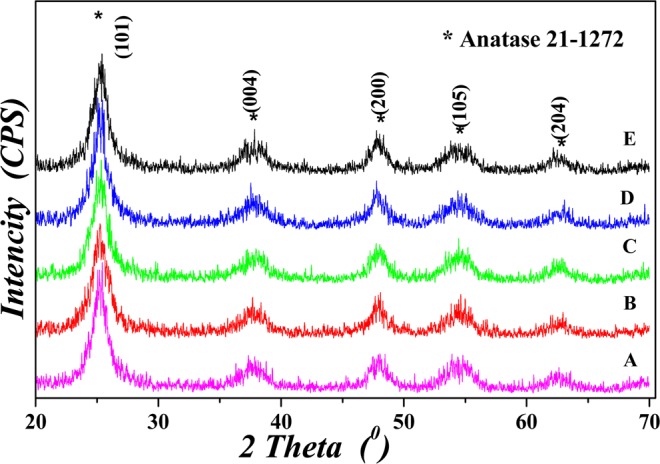
XRD patterns for the hydrated TiO_2_.

**Figure 2:**
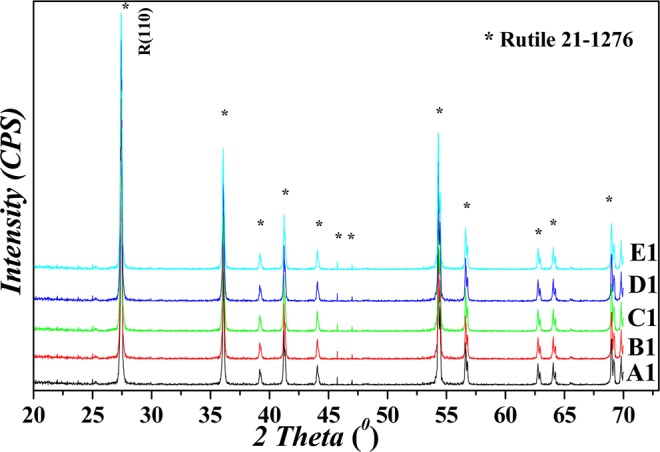
XRD patterns for the rutile TiO_2_ pigments.

The crystal structure of the obtained hydrated TiO_2_ had great impacts on the crystal structure of titanium dioxide pigment, and would ultimately affect the pigment properties of titanium dioxide. The XRD patterns of the as-prepared hydrated TiO_2_ series were showed in Fig. 1, and the XRD patterns for rutile TiO_2_ in Fig. 2. The crystal size for anatase *L*_*(101)*_ of hydrated TiO_2_, and the crystal size for rutile *L*_*(110)*_, the rutile content *X*_*R*_ and pigment properties for rutile TiO_2_ white pigment were listed in Table 1.

**Table 1:** Crystal size, rutile content and pigment properties for hydrated TiO_2_ and rutile TiO_2_ pigment.

Hydrated TiO_2_	L_(101)_(nm)	Rutile TiO_2_	X_R_(%)	L_(110)_(nm)	TCS	SCX	Jasn
A	8.42	A1	98.5	124.2	1780	2.25	94.54
B	8.14	B1	98.8	134.1	1820	2.56	94.86
C	7.83	C1	99.1	142.4	1840	3.18	95.17
D	7.57	D1	99.4	147.6	1810	2.64	94.93
E	7.10	E1	99.9	158.3	1700	2.19	94.68
R930	/	R930	/	/	1910	3.12	94.52

In the hydrolysis system to produce titanium dioxide pigment, the hydrolysis intermediates (also named hydrated TiO_2_, or metatitanic acid) would absorb a large amount of water and sulfate anion to form the crystalline structure with anatase phase, due to the presence of a large number of sulfate anion in the hydrolysis system, with the molecular structure as H_2_TiO_3_·H_2_O. In Fig. 1, all the XRD patterns of the hydrated TiO_2_ samples were clearly consistent with the standard anatase TiO_2_ phase (*JCPDS* 21–1272), without any other crystal phase diffraction peaks, showing with only the anatase phase. The hydrated TiO_2_ was anatase phase with low crystallinity due to their wide flat diffraction peaks and low diffraction intensity. The crystal size *L*_*(101*)_ for hydrated titanium dioxide ranged from 8.42 *nm* to 7.10 *nm*. Hydrated titanium dioxide was transformed into rutile TiO_2_ structure after salt treatment and calcinations, and the rutile crystal phase structure was consistent with the standard rutile TiO_2_ (*JCPDS* 21–1276). Due to the difference of crystal size, particle size distribution and impurity content for metatitanic acid, there was a small deviation in the rutile crystallization process during the calcination process, resulting in a small deviation in the XRD pattern. The rutile content (*X*_*R*_) increased gradually with the decreasing of the crystal size *L*_*(101)*_ of hydrated titanium dioxide (as listed in Table 1). The negative linear correlation between *X*_*R*_ and *L*_*(101)*_ were shown in Eq. (1). In the equation, ***R*** represented as the correlation coefficient and ***SD*** represented as the standard deviation.

1 $${X}_{R}=107.42\,-\,1.06\,\ast \,{L}_{(101)},R=0.9998,SD=0.01362$$

When the crystal size of hydrated TiO_2_ was small, it was easier to transform into rutile TiO_2_ crystalline by surface atom diffusion during calcination process due to its higher crystal surface energy. In the calcination process, the phase transformation from anatase to rutile was mainly through the surface atomic diffusion, in order to use the atomic reconstruction to reduce the energy of the crystal and form a stable rutile structure. When rutile crystal nucleus was formed, the TiO_2_ crystal ions continuously aggregated and grew on the newly formed rutile crystal nucleus through surface atom diffusion, which would make the rutile titanium dioxide crystal growing. And at the same conditions, the smaller crystal size of hydrated TiO_2_ needed the lower calcination conditions and strength for crystal phase transformation from anatase to rutile. In order to meet the requirement of rutile content for rutile TiO_2_ production (*X*_*R*_ > = 98%), the anatase crystal size of the hydrolyzed hydrated TiO_2_ should be controlled below 8.89 *nm*. However, when the crystal size of hydrated TiO_2_ was too small and with obvious colloidal properties, the calcined particles would be easily sintering to form larger rutile titanium dioxide particles, resulting in deteriorating the rutile TiO_2_ pigments properties.

The negative linear mathematical relationship of crystal size between *L*_*(110)*_ for the rutile TiO_2_ and *L*_*(101)*_ for the hydrated TiO_2_ was as the following, Eq. (2).

2 $${L}_{(110)}=338.79\,-\,25.28\,\ast \,{L}_{(101)},R=0.9943,SD=1.595$$

From Eq. (2), as crystal size decreasing of hydrated TiO_2_, it would be easy to transform hydrated TiO_2_ to rutile structure and promote the atomic diffusion in the calcination process basing on the high surface energy of hydrated TiO_2_, which would make the phase transformation and crystal growth of rutile TiO_2_ easier and obtain the larger rutile crystals. On the other hand, it would be easier to cause the sample sintering and agglomerate to form larger particles when the crystal size of hydrated TiO_2_ was too small.

The pigment properties of rutile TiO_2_ pigments (also named initial product) were mainly determined by its crystal structure, particle size and its distribution. The chromatic power (*TCS*) of the calcined rutile TiO_2_ products ranged from 1700 to 1840. Without coating post-treatment, the *TCS* values for the rutile samples were lower than the reference sample R930 which was with coating post-treatment. The *TCS* of the samples increased firstly and then decreased as the crystal size of hydrated TiO_2_ decreasing gradually. The mathematical relationship between *TCS* and *L*_*(101)*_ was as the following equation, Eq. (3).

3 $$TCS=-\,11415.26+3354.25\,\ast \,{L}_{(101)}\mbox{--}212.26\,\ast \,{{L}_{(101)}}^{2},{R}^{2}=0.9959,SD=4.983$$

Equation (3) showed quadratic linear relationship with high fitting degree. After taking the derivation, it showed that the maximum value of *TCS* was of 1836.2 (about 1840) when the crystal size of hydrated TiO_2_ was of 7.90 *nm*, indicating that the pigment properties of rutile TiO_2_ were partly determined by the structure of hydrated TiO_2_. And the crystal size of hydrated TiO_2_ could be controlled in an appropriate range by adjusting the hydrolysis conditions such as the number and quality of the hydrolysis seeds, concentration of TiOSO_4_ solution and hydrolysis time, which could effectively improve the product pigment properties. It could also approximately predict the pigment properties according to crystal size of hydrated TiO_2_ under certain conditions.

The blue phase (*SCX*) of the rutile pigment showed a similar changing trend as that of *TCS*, which was gradually increasing from 2.25 to 3.18, and then decreasing to 2.19. It was harmful for phase transformation from anatase to rutile and rutile crystal growth during calcination process when the crystal size of hydrated TiO_2_ was too large or too small. Because of the inconsistent phase transformation and growth of rutile crystal, it was easier to cause uneven growth of rutile TiO_2_ crystalline grain, ultimately reducing its sintering resistance and the *SCX* value. The brightness index (*Jasn*) of the calcined products was better and higher than that of the reference sample R930 (94.52). The *Jasn* also showed the same changing trend as that of *TCS*, increasing gradually to 95.17 at first and then decreasing to 94.68.

Appropriate crystal structure and suitable crystal size of hydrated TiO_2_ were helpful to promote the phase transformation from anatase to rutile and the rutile crystal growth during the calcination process to obtain good rutile TiO_2_ crystalline structure, resulting in proper crystal size of the rutile titanium dioxide. The integrity of rutile crystal structure ensured its high refractive index and scarcely any sintering particles, which could improve the calcination process and obtain rutile TiO_2_ with suitable particle size distribution and better crystal structure. All these influencing factors were helpful to improve the rutile pigment properties.

### Particle size distribution and impurity iron content

The particle size distribution (*PSD*) of the rutile titanium dioxide also had important influences on the pigment properties. Because the *TCS* of the rutile TiO_2_ was related to the light scattering coefficient (*S*) and the light absorption coefficient (*K*), the larger of *S* value and the smaller of *K* value, the larger of *TCS*, and the better of the covering power and whiteness of the titanium dioxide pigment^25^. Generally, in order to improve the *S* value, the particle size of the pigment should be controlled in the range of 0.15–0.35* µm* in the visible light range, and the amount and content of titanium dioxide particles should be maintained as higher as possible in the range about 0.2 *µm*. The average particle size (*D*_*AV*_), particle polydispersity (*Pdi*), relative scattering force (*R*_*S*_) and impurities content of the calcined rutile TiO_2_ pigments were showed in Table 2.

**Table 2:** Effect of particle size distribution and impurities content of hydrated TiO_2_ on rutile TiO_2_ pigment.

Hydrated TiO_2_	D_AV_ nm	Pdi	S_BET_ m^2^/g	Fe %	Rutile TiO_2_	D_AV_ nm	Pdi	Wt,% (0.15~0.30 µm)	Fe %	ZnO %	K_2_O + Na_2_O %	P_2_O_5_%	R_S_ %
A	1130	0.433	275	0.0042	A1	339	0.362	66.8	0.0040	0.2295	0.1522	0.1025	97.8
B	976	0.341	256	0.0022	B1	307	0.265	71.4	0.0021	0.2283	0.1519	0.1021	98.4
C	782	0.132	243	0.0012	C1	238	0.107	78.2	0.0011	0.2272	0.1526	0.1018	99.2
D	887	0.369	267	0.0034	D1	321	0.303	69.7	0.0033	0.2279	0.1531	0.1020	97.9
E	1029	0.475	282	0.0052	E1	372	0.387	64.1	0.0050	0.2284	0.1534	0.1022	97.1
R930	/	/		/	R930	/	/	/	/	/	/	/	100

The *D*_*AV*_ of hydrated TiO_2_ ranged from 0.782* µm* to 1.130 *µm*. The polydispersity (*Pdi*) was used to characterize the particle size distribution of the mono-dispersity particles, and the smaller the value was, the more concentrated particle size distribution was. The *Pdi* value was the index indicating the wide and narrow of particle size distribution, the smaller the *Pdi* value was, the narrower the *PSD* was. The *Pdi* of hydrated TiO_2_ ranged from 0.132 to 0.475, and sample C was the smallest with the narrowest *PSD*. The *D*_*AV*_ of the calcined rutile TiO_2_ ranged from 0.238 *µm* to 0.372 *µm*, with the *Pdi* value ranging from 0.107 to 0.387, of which sample C1 had the narrowest *PSD* with *Pdi* of 0.107. It showed that the *Pdi* value of titanium dioxide prepared by hydrated TiO_2_ with smaller *Pdi* value was also smaller than the others (Table 2), which indicated that narrower *PSD* of hydrated TiO_2_ was beneficial to obtain TiO_2_ powders with narrower *PSD*. The *Pdi* values for hydrated TiO_2_ and rutile TiO_2_ were met with the following mathematical relationships.

4 $$Pd{i}_{TiO2}=-\,0.00554+0.82955\,\ast \,Pd{i}_{MA},R=0.9975,SD=0.00893$$

The relationships of *Pdi* showed positive linear correlation (Eq. (4)). Narrower *PSD* of hydrated TiO_2_ was beneficial to control the crystal phase transformation and crystal growth of rutile TiO_2_ in a suitable uniform range during the calcination process. When the *PSD* of hydrated TiO_2_ was narrower, as the particle size was more uniform in the calcining process, and the properties and compositions of the active sites of anatase TiO_2_ in the phase transformation and crystal growth were closer, by atomic diffusion and crystal growth on the particle’s surface, the obtained calcined rutile particles were uniform. Ultimately, the obtained calcined rutile TiO_2_ was with narrower and more uniform *PSD*, which could effectively improve the pigment properties. While when the *PSD* of hydrated TiO_2_ became wider, the *PSD* of the calcined rutile TiO_2_ became wider due to the inconsistent diffusion process and crystal growth during the calcining process, which ultimately deteriorated the pigment properties. The mass content of titanium dioxide with particle size distribution in the range of 0.15–0.30* µm* was listed in Table 2. This further proved that when the *PSD* was narrower, the higher the mass ratio of particles in the suitable size range for rutile TiO_2_ products, the better the corresponding pigment properties. The relative scattering force (*Rs*) refers to the ratio of the scattering ability of a pigment to the incident light in a certain medium compared with the reference pigment, and the larger the ratio, the better the pigment performance. The relationship between *R*_*S*_ and *D*_*AV,TiO2*_ was as the following equation.

5 $${R}_{S}=102.96-0.01547\,\ast \,{D}_{AV,TiO2},R=0.9857,SD=0.1516$$

The relationship between *R*_*S*_ and *D*_*AV,TiO2*_ showed negative linear correlation (Eq. (5)). In order to improve the *R*_*S*_ value, it was necessary to control the product particle size near 0.20 *µm* calculated from Eq. (5) and maintain it in a narrow *PSD* range, so as to obtain rutile TiO_2_ with excellent pigment properties.

**Figure 3:**
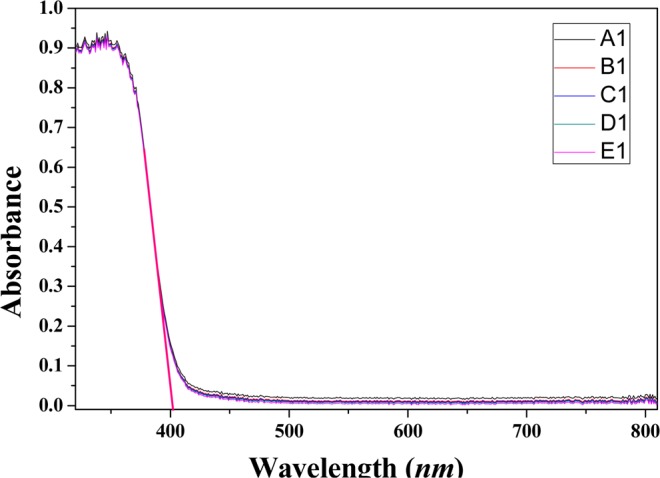
UV–vis diffuse reflection spectra for the rutile TiO_2_ pigments.

The UV–vis spectra for the rutile TiO_2_ pigments were showed in Fig. 3. The absorption spectra for all the samples were consistent, there was a strong absorption in the wavelength region less than 402.4 *nm*, and the difference of absorption intensity was not significant. This part of absorption corresponded to the intrinsic absorption of rutile titanium dioxide, which was the energy absorbed by the electron transition from the valence band of titanium dioxide to the conduction band (the intrinsic forbidden band width, 3.08 eV). The fine fluctuation of the absorption spectra might be caused by different sizes of the rutile crystals.

**Figure 4:**
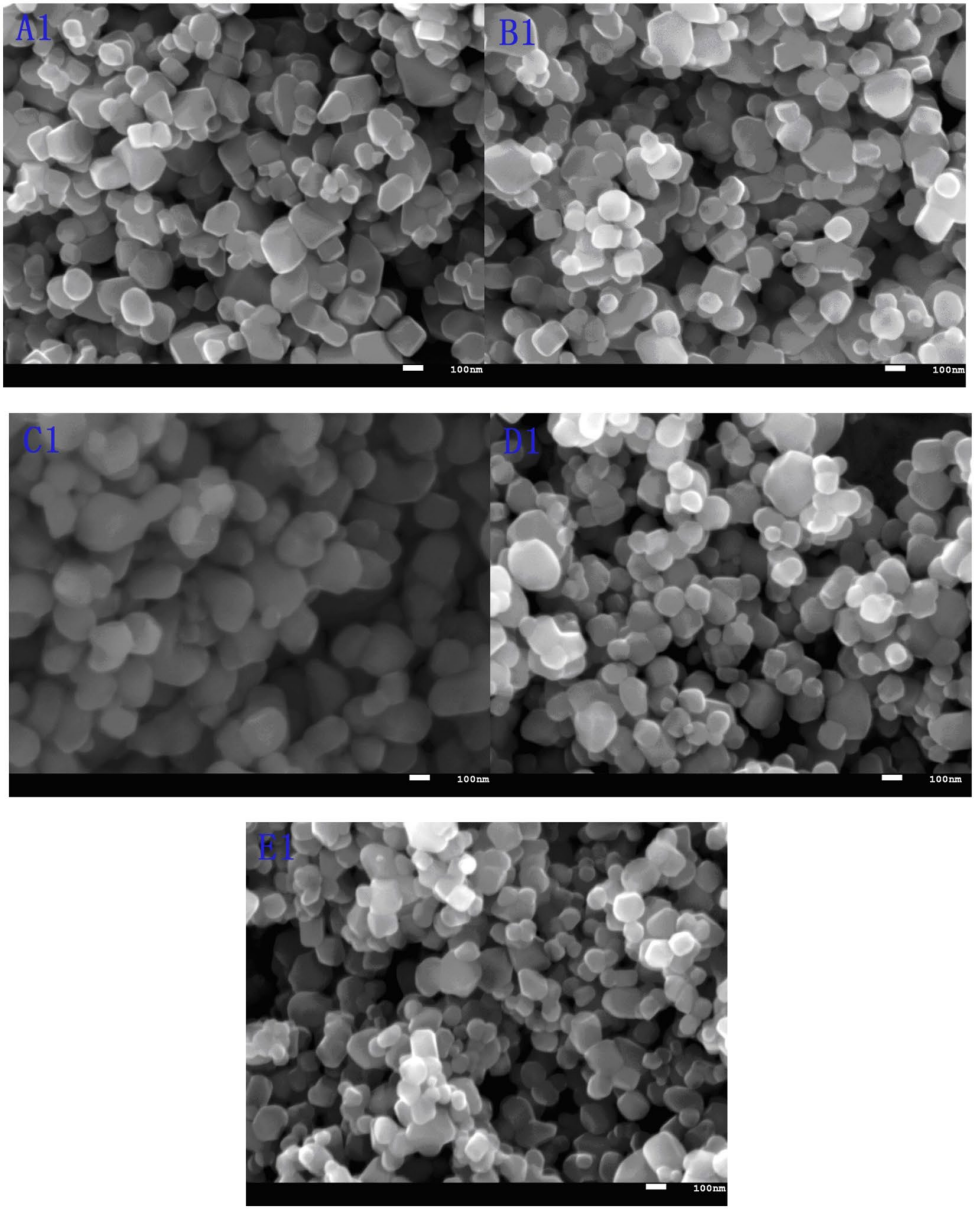
SEM photographs for rutile TiO_2_ pigment.

The SEM photographs for the rutile samples were shown in Fig. 4. All the particles showed rutile TiO_2_ morphologies with clearly crystal contour, and the high crystalline was consistent with the XRD analysis. The particle size ranges from 70 *nm* to 300 *nm*, and the main average particle size was of about 120 *nm*. During the calcination process, the smaller particles might congregate together to form a larger one due to re-crystallization because its higher surface energy and crystal growth drive, resulting in larger particle size and larger *D*_*AV*_, as showing in Table 2. It could be seen that the larger and wider the particle size of hydrated TiO_2_ was, the wider *PSD* of rutile TiO_2_ products was, resulting in poor pigment properties, as listed in Tables 1 and 2. And sample C1 was with the narrowest *PSD* and the smallest *Pdi*, and it confirmed that the appropriate *PSD* for the hydrated TiO_2_ would improve the crystal growth and *PSD* for rutile TiO_2_. This also proved that appropriate particle size and narrow *PSD* of hydrated TiO_2_ were conducive to obtaining narrower and uniform particle size distribution of rutile TiO_2_, and reducing sintering phenomena for the particles, which would lead to improve the pigment properties. The regular crystallographic perfection, good and complete morphology, proper *PSD* for TiO_2_ was all beneficial to improve pigment properties.

**Figure 5:**
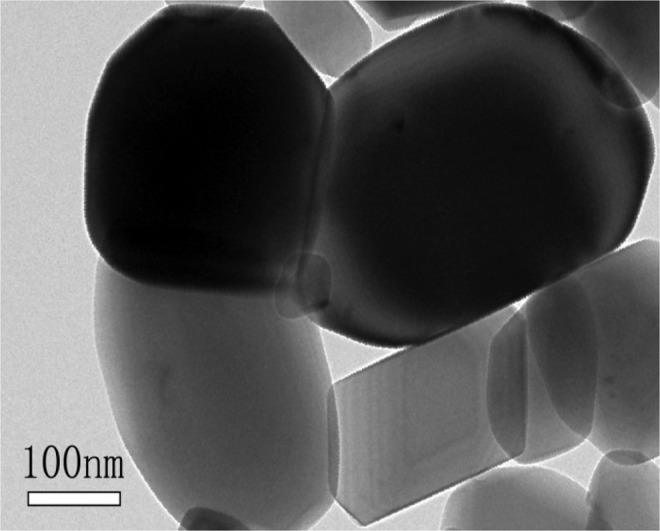
TEM photograph for sample C1.

The TEM photograph for sample C1 was showed in Fig. 5. The crystal profile of sample C1 was clear, and the particle uniformity was good. The average size was about 240 *nm*, which was consistent with the particle size test results. The size of particles was different, which might be related to the slow hydrolysis rate and uneven hydrolysis process of TiOSO_4_ solution. Better particle morphology and particle size distribution would contribute to the improvement of the pigment performances.

Because the hydrolyzed hydrated TiO_2_ was with small size of crystal and aggregates, large specific surface area and stronger colloidal properties, the impurities were easy to be adsorbed on and brought out by hydrated TiO_2_. As the adsorbed amount of colored impurities was one of the key factors affecting the structure of titanium dioxide, it was of great importance to improve pigment properties by controlling the adsorbed impurities in an appropriate range. The iron impurity was the main colored impurity, and when the content exceeded the limited range (commonly less than 30 *ppm* for rutile TiO_2_ and less than 90 *ppm* for anatase TiO_2_), it would cause the rutile samples yellowing and sintering, which would seriously worsen the pigment performances. The main impurities content including Fe, ZnO, K_2_O + Na_2_O and P_2_O_5_ of the rutile TiO_2_ pigments were listed in Table 2, and the content of these impurities was low, which had little effect on the properties of the rutile TiO_2_ pigments. The specific surface area (*S*_*BET*_) was a key factor that affecting the adsorbed amount of impurities. The absorbed content of iron ions gradually increased with the increasing of the *S*_*BET*_ for hydrated TiO_2_ (as showed in Table 2). The relationship between the adsorption amount of impurity iron (*% Fe*) and the *S*_*BET*_ of hydrated titanium dioxide was as the following equation.

6 $$ \% Fe=-\,0.02371+1.01839\,\ast \,{10}^{-4}\,\ast \,{S}_{BET},R=0.9948,SD=0.0001869$$

The *% Fe* had positive linear correlation with the *S*_*BET*_ of hydrated TiO_2_. The *S*_*BET*_ of the hydrated TiO_2_ should be as small as possible in order to control the *% Fe*. The *S*_*BET*_ of hydrated TiO_2_ was connected with the hydrolysis conditions and operational parameters. When the hydrolysis conditions were well controlled, the hydrolysis reaction was conducted more uniform, the precipitated hydrated TiO_2_ particles were more well-distributed and the formed aggregates were with narrower particle size distribution and relative smaller *S*_*BET*_. At the same time, the colloidal properties of hydrated TiO_2_ would be weakened by increasing the hydrolysis temperature and prolonging the hydrolysis time, which could reduce the *S*_*BET*_ and adsorption amount of impurity iron, resulting in better pigment properties. In addition, it was also great important that hydrated TiO_2_ with narrower particle size distribution and smaller *S*_*BET*_, which could be conducive to reduce the subsequent washing water consumption, shorten the washing time and reduce the washing strength and cost.

## Conclusions

The rutile TiO_2_ pigments were prepared through thermal hydrolysis by authigenic seed method via Short Sulfate Process. The structural factors such as crystal structure, particle size distribution, impurity content of the iron ion and specific surface area of the hydrated titanium dioxide had great important impacts on the crystal structure, pigment properties and *PSD* of the rutile TiO_2_, and there had also an internal influencing relationship among these factors, these factors influenced and determined each other. Suitable crystal size and crystal structure of the hydrolyzed hydrated TiO_2_ were helpful to promote the phase transformation from anatase to rutile and crystal growth of rutile TiO_2_, and it was also related to the rutile content, crystal size and pigment properties of rutile TiO_2_ satisfying with mathematical regression correlation. It was advisable to control the crystal size of hydrated to be less than 8.9 *nm* and close to 7.9 *nm*, which could obtain rutile TiO_2_ with good crystal structure which could reduce the sintering of the particles and enhance the pigment performances for rutile TiO_2_ pigments. The appropriate particle size and particle size distribution of hydrated TiO_2_ had obvious effects on the particle size distribution, polydispersity and relative scattering force of the rutile TiO_2_ particles. The adsorption amount of impurity iron ions was greatly affected by the *S*_*BET*_ of hydrated TiO_2_, and there was a correlation relationship between them. Rutile titanium dioxide pigments with good structure, regular morphology and excellent pigment properties could be prepared by controlling the hydrolysis conditions to obtain hydrated TiO_2_ with the particle size distribution as much narrower as possible, and lower impurity content of iron. Appropriate structural factors of hydrated TiO_2_ were helpful to prepare titanium dioxide with excellent pigment properties.
